# Alignment options and robotics in total knee arthroplasty

**DOI:** 10.3389/fsurg.2023.1106608

**Published:** 2023-02-09

**Authors:** Justin O. Aflatooni, Austin E. Wininger, Kwan J. Park, Stephen J. Incavo

**Affiliations:** Department of Orthopedic Surgery and Sports Medicine, Houston Methodist Hospital, Houston, TX, United States

**Keywords:** total knee arthoplasty, alignment, mechanical, kinematic, robotics, functional

## Abstract

Total knee arthroplasty is one of the most widely performed surgical procedures today. Its widespread popularity has helped drive innovation and improvement in the field. Different schools of thought have developed regarding the best way to perform this operation. Specifically, there are controversaries regarding the best alignment philosophy for the femoral and tibial components to optimize implant stability and longevity. Traditionally, neutral mechanical alignment has been the preferred alignment target. More recently, some surgeons advocate for alignment matching the patient's pre-arthritic anatomic alignment (“physiologic” varus or valgus), which has been described as kinematic alignment. Functional alignment is a hybrid technique that focuses on the coronal plane minimizing soft tissue releases. To date, there is no evidence demonstrating superiority of one method over another. There is growing popularity of robotic surgical techniques to improve accuracy of implant position and alignment. The choice of alignment philosophy is an important aspect of robotic assisted TKA surgery and has the potential to clarify the optimal alignment technique.

## Introduction

Total knee arthroplasty (TKA) is a commonly performed orthopedic procedure with generally good outcomes. However, since its inception, differing schools of thought have developed regarding the best approach for reconstructing, aligning, and producing a balanced knee. In this paper, we will review the current concepts in TKA alignment as well as robotic-assisted surgical methods.

## Mechanical alignment

Mechanical alignment is the approach whereby a knee is reconstructed with an intended goal of a hip-knee-ankle (HKA) angle of 180° by positioning both the tibial and femoral components perpendicular to the mechanical axis of each bone. In this method, coronal alignment is prioritized, and the same bone cuts are made in every knee, regardless of unique patient characteristics or deformity. This philosophy is backed by engineering principles that neutral limb mechanical alignment results in equal mechanical load on implants to avoid early prosthesis/polyethylene wear and tibial component loosening (1, 2).

Mechanical alignment is classically performed with a 5–6° valgus femur cut as measured with conventional instrumentation, which is most often intramedullary, that represents the anatomic axis. Newer methods to determine the mechanical axis include navigation, patient specific instrumentation, and robotic-assisted techniques. For the tibia, an extramedullary guide is commonly used to perform a perpendicular cut (90°) to the long axis (representing both the anatomic and mechanical axis). With the knee in flexion, rotation of the femoral component is established to make a posterior femoral cut that is parallel to the tibial cut and to create a rectangular flexion space. Femoral rotation targets can be achieved with measured resection or gap balancing techniques, which differ in the degree and timing of soft tissue releases (3).

### Measured resection

Measured resection makes all bony cuts based on anatomic landmarks first, followed by soft tissue releases as needed to create symmetric flexion and extension gaps. This technique utilizes the posterior femoral condylar axis (PCA), anteroposterior (AP) axis, and/or trans-epicondylar axis (TEA) to gauge femoral rotation, and bony resections are typically made irrespective of soft tissue tension. Rotational positioning of the femoral component parallel to the TEA is associated with decreased femoral lift-off (4). Furthermore, the TEA does not significantly change with degenerative deformity or revision cases, and thus does not lose reliability as a landmark in these situations (3). However, some authors have noted that intraoperative identification of the TEA can be inconsistent even among experienced surgeons (5–7). The AP axis (Whiteside's line) line runs through the crest of the trochlea and intersects the middle of the posterior condylar axis. Although the AP axis can help determine femoral rotation, this line depends on normal trochlear anatomy. Thus, this method can prove inaccurate in the presence of femoral dysplasia or significant degenerative changes, which may result in internal rotation of the femoral component (8). The PCA can also be used to determine femoral rotation (arbitrarily sets external rotation at 3–5° relative to PCA), though this is reliant on normal anatomy and thus may not be appropriate for all cases (9, 10).

### Gap balancing

Gap balancing utilizes the patient's soft tissue (medial/lateral collateral ligament) tension to determine bony resection. Proponents of a gap balancing technique argue this technique may lead to better coronal plane stability from extension to deep flexion.

Historically, gap balancing was achieved by first focusing on the flexion gap (11). In the flexion gap first method, a perpendicular proximal tibial resection, relative to the longitudinal axis of the tibia, is the first bony resection. A flexion gap tensioning jig is used to assure appropriate flexion gap symmetry (proximal tibia is parallel to the TEA) prior to making the posterior femoral condylar resection. Attention is then turned to the extension gap and tensioning devices that are set to the same tension level to the flexion gap are used to guide the distal femoral resection.

Alternatively, to minimize the risk of joint line alteration, a gap balancing technique that focuses on the extension gap first has been described and is now more commonly utilized (12). The distal femur resection (5–7° valgus) is performed with an intramedullary guide, followed by the proximal tibial resection perpendicular to the longitudinal tibial axis. A spacer block or tensioner can then be used to evaluate ligamentous structures that need to be released to achieve neutral limb alignment and a symmetric extension gap. Focus is then turned to the flexion gap. If the knee is well balanced in extension and the proximal tibial resection is accurate, the TEA should be parallel to the proximal tibia at 90 degrees of flexion. An implant specific tensioner, laminar spreader, or spacer block is used to equally tension the collateral ligaments with the knee at 90° of flexion to determine the rotation and magnitude of the posterior femoral condylar resection. This technique prioritizes an equal and rectangular flexion and extension gap.

### Pitfalls

There is a paucity of evidence to suggest any significant clinical or functional difference between measured resection and gap balancing (13, 14). Gap balancing has been hypothesized to achieve better soft tissue balance due to this technique not relying on bone landmark identification with the knee in flexion (15). Furthermore, some studies attest that there is less femoral condylar lift-off (rectangular flexion gap) with the gap balancing approach than with the measured resection method throughout knee range of motion (16). Still, other studies indicate that there may be more soft tissue release, bone resection and joint line elevation with gap balancing, while the measured resection technique may have unequal medial/lateral (i.e., not rectangular) extension gaps (17).

Although restoration of neutral mechanical alignment is a reproducible goal during total knee arthroplasty, the philosophy of mechanical alignment is not without shortcomings. Making the distal femoral cut with 6° of valgus is based on this angle most often being in the range of 5–7° but does not account for patient specific anatomy. For example, shorter patients may need a distal femur resection of more than 6°, while 5° or less may be optimal for taller patients (18). Moreover, the intramedullary canal method to determine femoral anatomic alignment can result in substantial malalignment errors if minor malposition of the intramedullary rod occurs due variables such as canal diameter, starting hole orientation, rod length and width dimensions, or unique patient anatomy (19). Although asymmetric bearing wear is theoretically minimized with neutral mechanical alignment, component positioning to achieve this target may affect native femoral flexion axis, ligament tension, quadriceps function, and patella tracking (20, 21). Finally, if the patient has pre-arthritis varus or valgus, mechanical alignment will always require ligament balancing/releasing to achieve medial-lateral stability.

## Kinematic (anatomic) alignment

Anatomic alignment is considered the precursor of kinematic alignment and intends to recreate the anatomic (6–7°) femoral valgus and anatomic (3°) tibial varus appreciated in normal knee anatomy (22). The rotation of the femoral component is set to the posterior condylar axis. Prior to navigated or robotic-assisted surgery, the reproducibility of performing precise valgus and varus cuts was called into question.

Kinematic alignment assumes the non-arthritic knee is perfectly balanced and attempts to restore the native anatomy of the knee to restore the native balance of the soft tissue envelope (23). The goal of kinematic alignment is to recreate or resurface the pre-arthritis anatomy to achieve symmetric balanced extension, medial pivot throughout arc, lateral laxity in flexion, and central patellar tracking. Typically, calipered bone resection is performed to take away only as much bone and cartilage as is replaced with metal and polyethylene (adjusted for bone/cartilage wear, saw blade thickness, and osteophytes). The goal is to perform no soft tissue releases and to achieve normal knee kinematics.

Kinematic alignment is supported with more recent data demonstrating that there is a large percentage of the normal population (32% of men, 17% of women) with constitutional varus and a HKA alignment of 3° or more at skeletal maturity (24). A particular benefit of kinematic alignment includes the avoidance of ligamentous releases to achieve medial-lateral balance in extension. In patients with constitutional varus and a varus joint line orientation, attempting to achieve a neutral mechanical axis may alter the natural joint line (25). In the sagittal plane, the tibial component is positioned such that the posterior tibial slope matches the flexion-extension plane of the native joint.

Kinematic alignment has been reported to achieve a neutrally aligned knee with no need for balancing the flexion gap (26) and, if properly executed, there are no differences in clinical or radiographic outcomes between this method and mechanically aligned TKAs (27). The reproducibility of a kinematically aligned TKA has been called into question by many arthroplasty surgeons and how well tolerated an oblique joint line is for a prosthetic knee is unknown and has been described as a possible downside (28). Inadvertently (*via* bone cut margin of error) creating too much tibial varus and/or femoral valgus can cause an overly oblique joint line. If natural varus is restored without sufficient external rotation of the femoral component, the patient may experience patellofemoral maltracking through increased contact stresses at the patellofemoral joint. Many surgeons do not agree that the flexion gap does not require balancing and are concerned that excessive lateral laxity in flexion can lead to flexion instability, femoral condylar lift-off, eccentric polyethylene loading, and premature loosening of the implants (29). Lastly, excessive posterior slope of the tibial component has been a reported risk factor for posterior subsidence of the tibial baseplate or excessive posterior edge wear of the polyethylene (30).

## Functional alignment

The authors strive to gain the advantages and avoid the shortcomings of both the kinematic and mechanical alignment schools of thought to achieve a functionally aligned TKA. Functional alignment emphasizes the coronal plane, but components are positioned in a manner that compromises the soft tissue envelope of the knee as little as possible. Thus, the soft tissues dictate the plane of the joint line and soft tissue releases are typically not needed. Distal femur and proximal tibia resection can be achieved with valgus and varus correction, respectively.

In functional alignment, the surgeon aims to recreate the patient's pre-arthritic condition by using gap balancing techniques to first create a rectangular extension space with the knee at 0° by first resecting distal femur in 3–5° of valgus and then the proximal tibia. Less valgus (3–5°) of the femoral cut eliminates the typical lateral laxity with 5–7° of valgus. This is because the native knee has lateral laxity to allow for the screw home mechanism in extension and for the convexity of the lateral tibial plateau as the knee flexes. The prosthetic knee has neither of these, so less femoral valgus will tighten and stabilize the lateral side, producing balance of the medial and lateral collateral ligaments without release.

**Figure 1:**
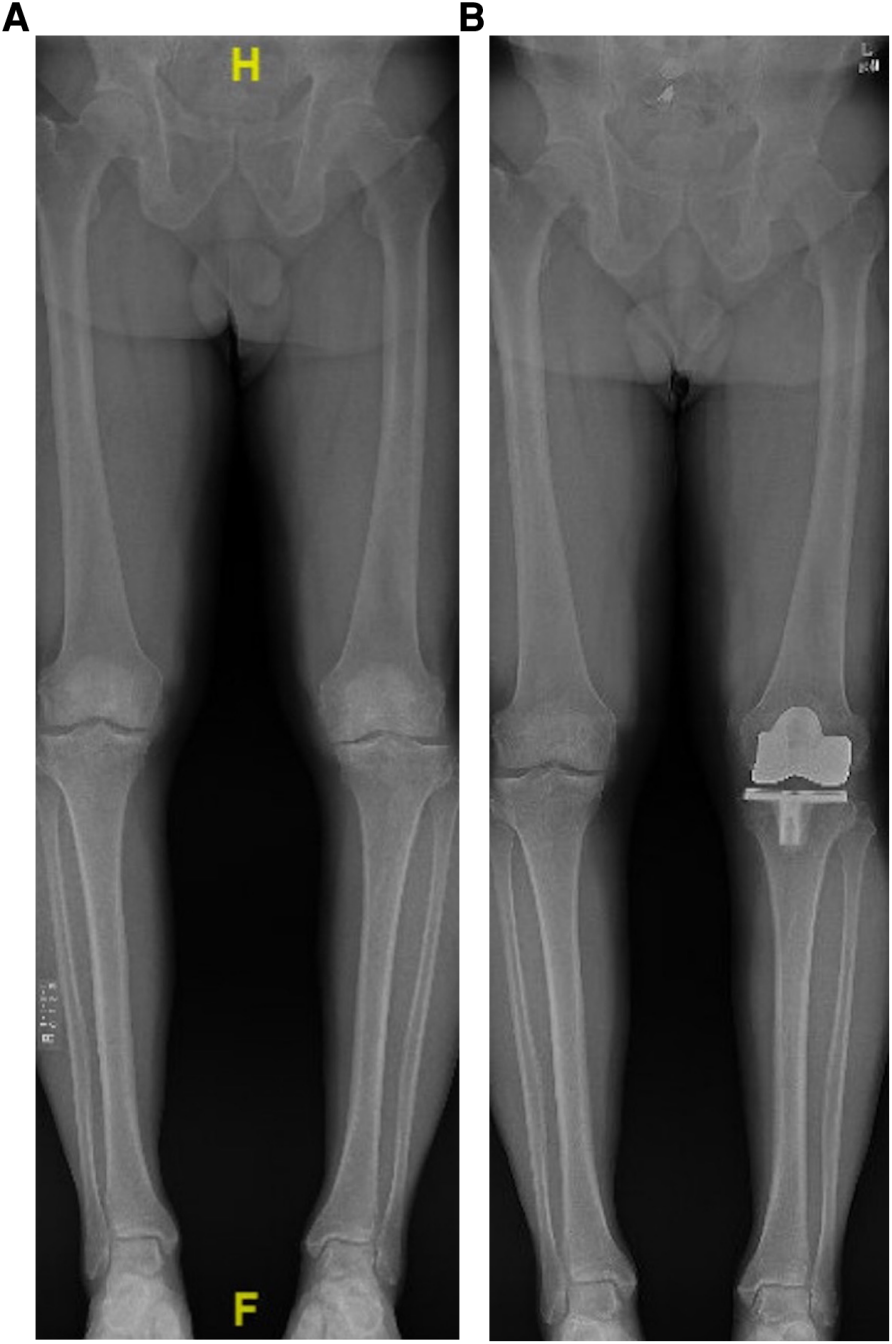
(**A**) example of pre-operative standing long-leg radiograph showing constitutional varus of the right lower extremity and varus malalignment of the left lower extremity secondary to end-stage osteoarthritis. (**B**) Post-operative standing long-leg leg radiograph using functional alignment to achieve slight varus alignment without any ligamentous releases.

**Figure 2:**
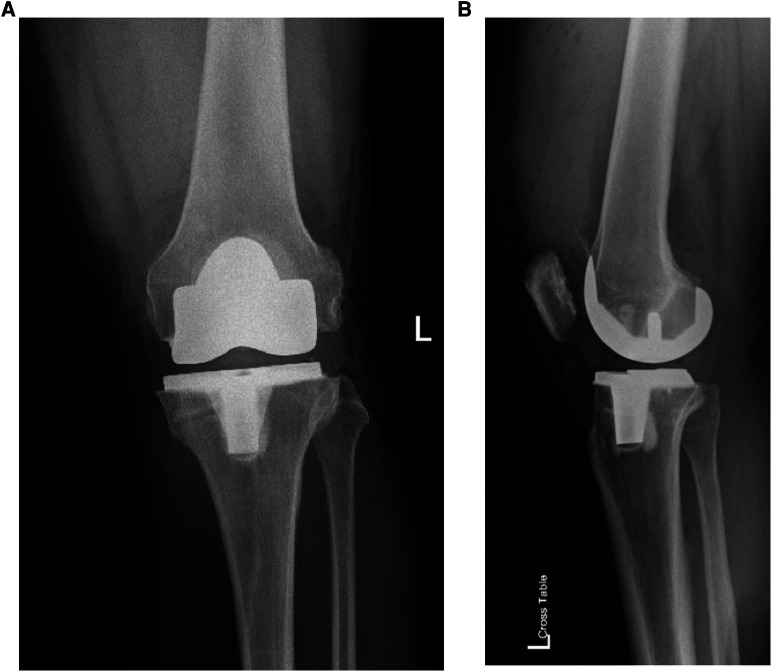
(**A**) anteroposterior and (**B**) lateral radiograph of the same patient demonstrates a balanced left TKA.

Preparation of the extension space is followed by creating a balanced flexion space at 90°. By aiming for a rectangular flexion gap, even when accounting for the margin of cutting error, the surgeon can reduce the risk of inadvertent internal femoral component rotation and creation of a pathologic Q-angle. By ensuring a balanced rectangular flexion gap, the surgeon can avoid further ligamentous release. This has clinical relevance as the addition of soft tissue releases after bony cuts may lead to worse patient-reported outcomes (31). Anecdotally, the authors have found that functional alignment consistently provides the patient with a stable and functional knee without ligamentous release in most cases (Figures 1, 2). The authors strive for a HKA angle of 2° of varus for all cases unless there is pre-operative valgus >2°, in which case the target HKA angle is 2° of valgus.

Opponents of natural alignment argue that aligning the components in varus (or valgus) places excess load on half of the knee joint. Whether this varus alignment alters patient outcomes, results in polyethylene catastrophic failure, or tibial loosening is not fully elucidated (23, 32).

## Robotics in total knee arthroplasty

Computer guidance has been adopted by arthroplasty surgeons to control alignment issues, to plan for complex deformity or prior hardware, to assist when patient body habitus alters landmarks, to determine flexion/extension gaps, and to increase accuracy of bony resection. Robotic systems in arthroplasty surgery include robotic arm-assisted procedures, robot-guided cutting jigs, and robotic milling using a range of passive to active systems (33). Passive robotic systems provide positional guidance for bony resection and implant positioning but lack haptic feedback. Semi-active robotic systems enable the surgeon to guide a robotic arm to perform bony resections within the confines of haptic restraint but require constant input from the surgeon. Active robotic systems operate independently to perform bony resections without real-time guidance after the surgical approach is performed and retractors are placed. There are image-dependent systems that rely on preoperative computed tomography (CT) or magnetic resonance imaging (MRI) scans. There are also imageless systems that require a more detailed intraoperative registration of bony anatomy (handheld probes create the topography of the knee) and joint kinematics. A benefit of these techniques is not using an intramedullary femoral guide to establish the anatomic axis for distal femoral resection.

Robotic-assisted techniques were developed to increase the precision of prosthetic alignment and have evolved to include soft tissue balancing algorithms (34). Tracking arrays are used to determine pre-resection flexion/extension and varus/valgus knee kinematics. In combination with a robotic ligament tensioning tool, this technology can accurately predict postoperative flexion and extension gaps prior to femoral resection (35). Ultimately, this may lead to a reduced need to perform soft tissue releases. With robotic-assisted techniques, the surgeon can achieve component position and non-neutral limb alignment targets more reproducibly with reduced alignment outliers (36–38). These techniques can be utilized for varying alignment goals (39). This enables the surgeon to better plan both component positioning and final polyethylene insert thickness than when compared to using a manual technique (40).

Arguments against robotics include the potential need for advanced preoperative imaging, added surgical time, integration into surgical workflow, and unknown cost-effectiveness. The risk for periprosthetic pin site fracture has been greatly reduced by using unicortical (instead of bicortical) pins and smaller diameter pins for tracking arrays. The clinical benefit of robotic-assisted techniques is yet to be determined and they have not been universally adopted by surgeons (41, 42). Augmented reality is an emerging technology in arthroplasty surgery that involves a computer-generated image that is superimposed on the surgeon's field of vision. This enabling technology can be achieved through a helmet attachment that can improve planning and execution of a procedure while allowing the surgeon to keep their eyes on the patient (43).

## Discussion

Several methods are described for surgically achieving a well-balanced knee, however, to date, no one school of thought has proven superior regarding patient outcomes. This is exemplified by a randomized controlled trial of simultaneous gap-balancing TKA and measured-resection TKA in 50 patients with bilateral varus alignment, in which there was no statistical difference in patient-reported or functional outcomes between the two methods of knee balancing (14). As previously stated, benefits and drawbacks exist for each described method of knee balancing. Not surprisingly, patient satisfaction may be able to be achieved with accuracy of measurable TKA variables within a precise range of measurements or techniques instead of a single, ultimate method or measurement. Preserving soft tissue stability, maintaining joint line orientation/height, maintaining HKA axis alignment within certain parameters, and installing implants within acceptable ranges are likely the most important factors for patient satisfaction and implant longevity.

## Conclusion

Alignment, or malalignment, in TKA can be a potential cause of patient dissatisfaction. When considering the optimal alignment target for implanting a TKA prosthesis, the relationship between the prosthesis and the soft tissue envelope may be more important than neutral mechanical alignment. Functional alignment represents a compromise between mechanical and kinematic alignment, and we believe represent the best of both techniques. It is our preferred method that leaves constitutional varus/valgus and sets rotation at 90° of flexion and avoids most ligament releases. However, regardless of the technique used, the goal remains to achieve a stable, functioning knee arthroplasty.
